# Transactions of the Medical and Chirurgical Faculty of Md.

**Published:** 1875-10

**Authors:** 


TRANSACTIONS OF THE MEDICAL AND CHIRURGICAL FACULTY OF MARYLAND. Seventy seventh Annual Session. Baltimore: April, 1875; pp. 226.

These transactions are divided into sections of Anatomy, Physiology, Gynecology, etc., and are gotten up with taste, and reflect credit both on the conbributors and publishing committee.

